# Evaluation of Treatment Protocols in Surgically Assisted Rapid Maxillary Expansion by Finite Element Analysis

**DOI:** 10.3390/medicina60091400

**Published:** 2024-08-26

**Authors:** Duygu Cihaner, Derya Karabulut, Ozen Dogan Onur, Erol Cansiz, Yunus Ziya Arslan

**Affiliations:** 1 Department of Oral and Maxillofacial Surgery, Faculty of Dentistry, Nisantasi University, Sariyer 34398, Turkey; duygu.cihaner@nisantasi.edu.tr; 2 Department of Mechanical Engineering, Faculty of Engineering, Istanbul University-Cerrahpasa, Avcilar 34320, Turkey; deryakarabulut@istanbul.edu.tr; 3 Department of Oral and Maxillofacial Surgery, Faculty of Dentistry, Istanbul University, Capa 34093, Turkey; ozend@istanbul.edu.tr; 4 Department of Oral and Maxillofacial Surgery, Istanbul Faculty of Medicine, Istanbul University, Istanbul 34452, Turkey; erolca@yahoo.com; 5 Department of Robotics and Intelligent Systems, The Institute of the Graduate Studies in Science and Engineering, Turkish-German University, Beykoz 34820, Turkey

**Keywords:** finite elements analysis, Le Fort corticotomy, separation of pterygomaxillary junction, surgically assisted rapid maxillary expansion, transverse maxillary deficiency

## Abstract

*Background and Objectives*: Transverse maxillary deficiency is an important maxillary anomaly that is very common in society and remains current in orthodontics. The maxillary expansion has been used in treatment for a long time. While maxillary expansion can be performed with rapid maxillary expansion in young adults, it is performed with surgically assisted rapid maxillary expansion (SARME) in individuals who have reached skeletal maturity. No consensus has been reached on the most successful surgical technique or the ideal appliance for treating transverse maxillary deficiency. Accordingly, we aimed to evaluate various surgical techniques and orthodontic appliances for treating transverse maxillary deficiency using the finite element method (FEM) to identify the treatment protocol that minimizes stress on the maxillary bone and teeth. *Materials and Methods*: On the virtual models obtained from the cone beam computed tomography of a patient, two different incisions (the pterygomaxillary junction is separated and not separated) were made and combined using three different orthodontic appliances (tooth, bone, and hybrid assisted). Then, stresses over the maxillary bone and maxillary teeth were calculated by FEM. *Results*: Our results showed that when the pterygomaxillary plates were separated, fewer stresses were observed on the bone and teeth. Although hybrid-supported appliances created less stress on the teeth than tooth-supported appliances and no difference was found between bone-supported appliances, it was found that hybrid-supported appliances created less stress on the bone than the other appliances. *Conclusions*: The separation of the pterygomaxillary junction in the SARME operation and the use of a bone-supported or hybrid-supported appliance would place less stress on the bone and teeth.

## 1. Introduction

Transverse maxillary deficiency is one of the maxillofacial deformities characterized by deep palate dome, maxillary stenosis, malocclusion, and crowding of teeth [1,2,3]. Congenital, iatrogenic, traumatic, genetic, and environmental factors may be effective in the etiology of maxillofacial deformities [1,3]. The demand for the surgical treatment of these deformities is coming from wider patient masses every day.

In individuals with transverse maxillary deficiency, the sutura palatina mediana should be separated and the upper tooth arch should be widened to correct the dental crowding. Since the closure of the midpalatal suture in humans has spread over a wide period, such as between the ages of 16 and 35, widening treatment can be performed using the rapid maxillary expansion (RME) method with orthodontic treatment in young adults. In individuals who have reached skeletal maturity, expansion of the maxillary arch is performed with surgical-assisted rapid maxillary expansion (SARME), which is a combination of surgery and orthodontic treatment, since RME will be insufficient due to age-related midpalatal suture resistance and the possibility of complications [4,5,6,7]. For determination of skeletal maturity, orthodontists use various radiographs that may include low-dose hand-wrist radiographs, occlusal and palatal radiographs, or cone-beam computed tomography if radiation dose is not a concern [4,5,6,7]. SARME surgery aims to expand the maxilla with the help of osteotomies that will reduce the resistance to the force to be applied for expansion due to the fibro-osseous junction of the facial and skull joints [2]. Osteotomies are applied to the edges of the aperture piriformis, the processus zygomaticus, the processus pterygoideus, and the sutura palatina mediana, where the upper jaw is resistant [5,7,8,9,10]. Since complications such as root resorption, only dental movement, lateral tipping of teeth, tooth extrusion, buccal cortical fenestration, palatal mucosal necrosis, and orthodontic recurrence can be seen due to RME treatment in individuals who have reached skeletal maturity, the SARME operation is a safe and simple surgical method that can be applied to these individuals, reducing the risk of recurrence [4,6,11]. Although SARME operations were first described in 1938, there is still no consensus on surgical application techniques [8]. Different incision techniques and different appliance designs have been used in the SARME operation from the past to the present to increase the clinical success rate. The age of the patient, the need for treatment, and the level of stress that may occur over the jaw in the application of orthodontic appliances can change the application protocol of the surgical operation [8]. The surgical techniques applied for maxillary expansion and the forces created by orthodontic appliances create stresses on teeth, bones, and adjacent anatomical structures [12].

In the treatment of RME, tissue stress can be evaluated using the finite element method (FEM) [13]. Most studies on SARME operations used clinical and cephalometric measurements to compare surgical techniques and appliances; however, there are few FEM studies [7,8,12,14]. No consensus has been reached on the most successful surgical technique or the ideal appliance for treating transverse maxillary deficiency [6]. Although some FEM studies investigated stresses in SARME, none compared surgical techniques across all three orthodontic expansion appliance types. Additionally, few studies detailed the stresses on the teeth [15]. To reduce stress-related complications post-SARME, the stresses created by orthodontic appliances in the surgical technique need analysis. The purpose of this study was to objectively evaluate different surgical techniques and orthodontic appliances (tooth-supported, bone-supported, and hybrid-supported appliances) for treating transverse maxillary deficiency. By using the FEM, this study aimed to analyze stress distribution on the maxillary bone and teeth, providing insights into which treatment protocols may minimize stress and improve patient outcomes.

## 2. Materials and Methods

**Figure 1:**
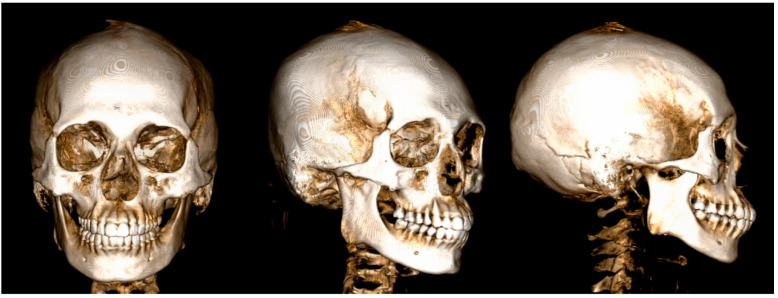
Three-dimensional views of the computed tomography data of the patient.

Three-dimensional (3D) cone beam computed tomography (CBCT) data of a 24-year-old male patient who needed a SARME operation due to transverse maxillary deficiency were used from the archive of the Department of Oral and Maxillofacial Surgery at Istanbul University, Faculty of Dentistry. Three-dimensional views of the tomography data are given in Figure 1. Intraoral examinations conducted prior to the study confirmed that the patient had no periodontal issues, indicating a periodontally healthy condition. The finite element analysis of the study was carried out at Istanbul University-Cerrahpasa, Faculty of Engineering, Department of Mechanical Engineering. Ethical committee approval was obtained from the Istanbul University Faculty of Dentistry Clinical Research Ethics Committee (Approval number: 13.12.2018/344). Written informed consent was obtained from the patient for permission to use the raw data of the patient’s CBCT.

### 2.1. Study Groups

In the study, the stress values over the maxilla and maxillary teeth were calculated by FEM after the SARME operation was virtually applied to the six groups shown in Table 1. Surgical techniques (the pterygomaxillary junction is separated and not separated) were divided into two main groups and each orthodontic apparatus (tooth, bone, and hybrid assisted) to be applied formed subgroups of the main groups (Table 1).

### 2.2. Modeling of the Maxilla and Teeth Models

The 3D models of the maxilla and teeth were generated by using the CT images (SCANORA3D, Soredex, WI, USA). The two-dimensional CT data were transformed into 3D maxilla and teeth models by employing an image processing program (3D-DOCTOR; Able Software Corp., Lexington, MA, USA). To differentiate the bone tissues from soft tissues, the Hounsfield unit (HU) threshold value for bone structure was established (upper limit: 3071 HU and lower limit: 226 HU) [16]. Then, computer-aided design software was used to obtain 3D solid models of the maxilla and teeth (Solidworks, Dassault Systemes SolidWorks Corp., Waltham, MA, USA). The maxilla and teeth models were obtained and created separately.

**Figure 2:**
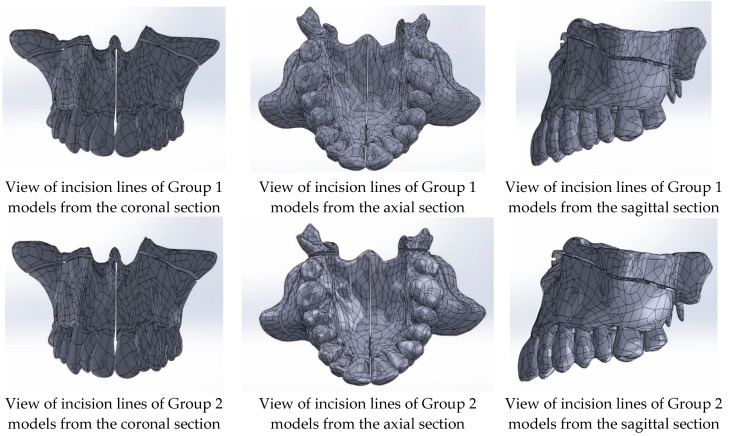
Incision lines of Group 1 and Group 2 models.

Once the model assembly was completed, incision lines for two predefined groups were created (Figure 2). For Group 1, midpalatal suture separation and Le Fort I corticotomy incision lines were generated. For Group 2, the pterygomaxillary junction separation was added to the midpalatal suture separation and Le Fort I corticotomy. The incision lines on the maxilla were planned to be 1 mm wide.

### 2.3. Finite Element Analysis

**Figure 3:**
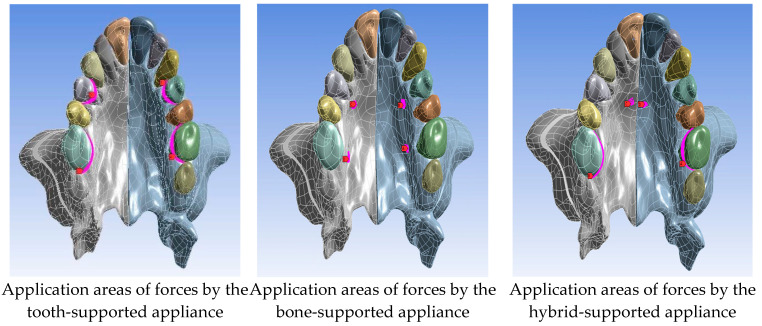
Force application areas in Group 1 and Group 2.

In our study, fixed points as the boundary conditions were determined as maxillary sinus medial wall and pterygoid plates. The applied forces were applied perpendicular to the selected areas on the surface of the bones and teeth according to the design of the appliance (Figure 3). In the figure, the pink areas show regions where forces are applied, while red arrows qualitatively show how much force is applied.

The magnitude of the force to be applied to the models was found by calculating the force required to open the midpalatal suture by 1 mm (Table 2). To expand the maxillary by 1 mm, the greatest force, 480 N was required in Model 1B (the model in which a bone-assisted appliance was applied and pterygoid plates are not separated). Then the second greatest force was required in Model 1C (the hybrid appliance model), and the least force, 325 N was required in Model 2A (the model in which a tooth-assisted appliance was applied and pterygoid plates were separated).

The models were then transferred to a finite element analysis software, ANSYS (ANSYS Inc., Canonsburg, PA, USA). The created 3D finite element model is the same for all groups. In ANSYS, von Mises stresses over the maxilla and teeth in each group were calculated. To perform finite element analysis, the biomechanical properties of the materials given in Table 3 were introduced.

The models were then meshed with the tetrahedral element. The mesh sizes were 1 mm for the maxilla and 0.5 mm for the teeth to increase the resolution of the simulation. The number of elements and nodes of the models are given in Table 4.

## 3. Results

**Figure 4:**
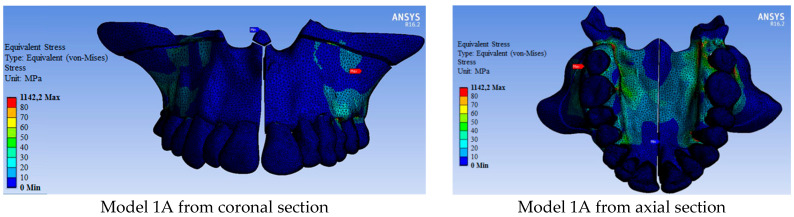

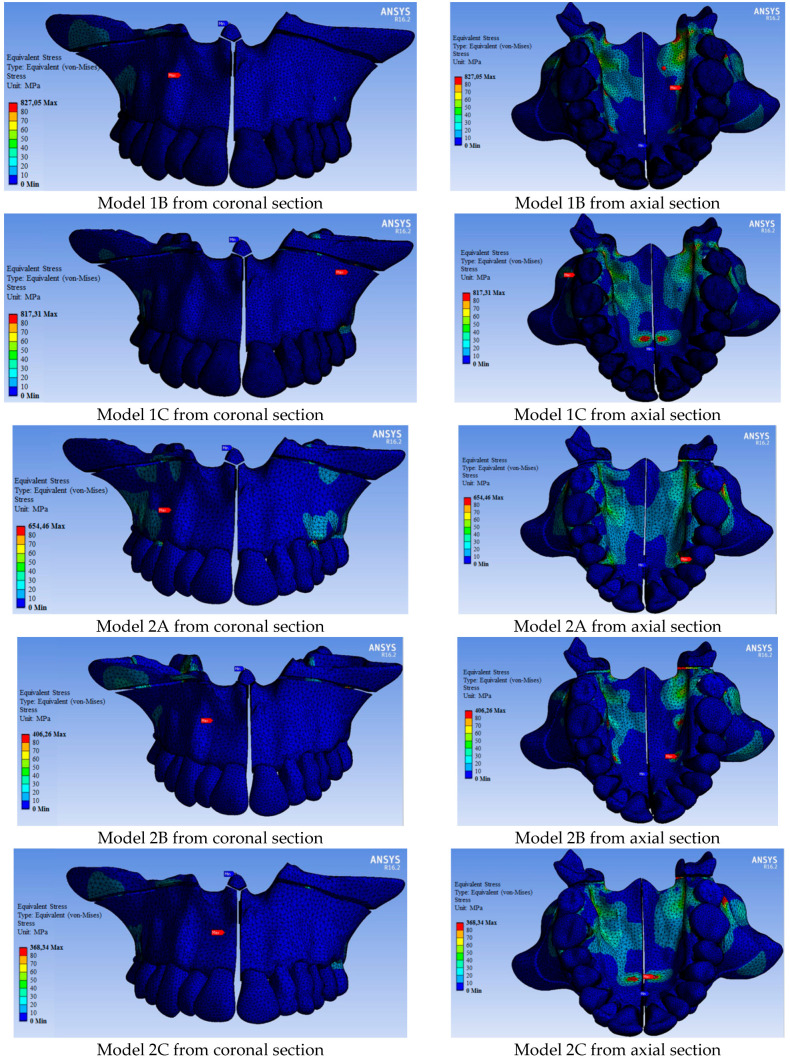
Distribution of von Mises stresses over the bone tissue (MPa).

Since the pterygoid plates were not separated in Group 1 (models 1A, 1B, and 1C), the maxilla was opened in the form of a ‘V’ and more stresses occurred in the posterior region (Figure 4). In Group 2 (models 2A, 2B, and 2C), a parallel opening occurred in the maxillary segments as the pterygoid plates were separated. The stresses in the posterior region were less in Group 2 compared to Group 1 models (Figure 4).

**Figure 5:**
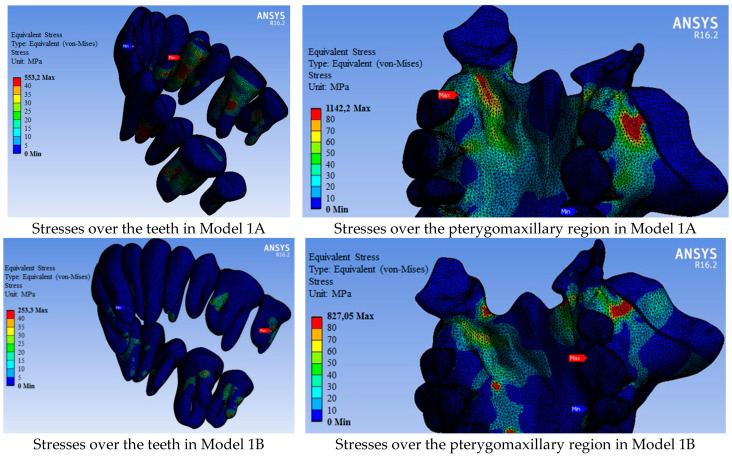

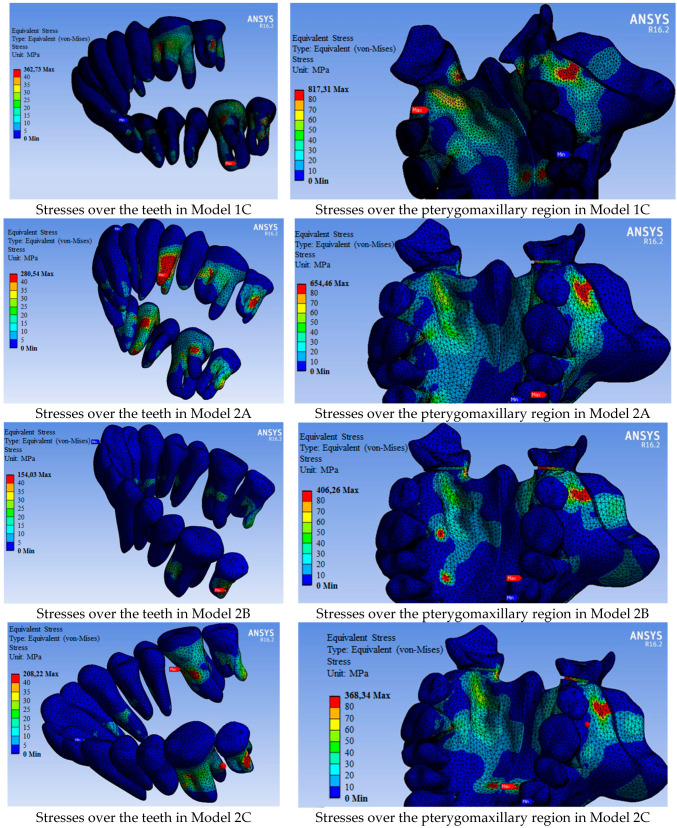
Von Mises stress values over the dental arc and pterygomaxillary region (MPa).

In general, more force was required in Group 1 models where pterygoid plates were not separated to expand the maxillary than in Group 2 models (Table 2). Accordingly, after maxillary expansion, more stress occurred in Group 1 models than in Group 2 models. (Figure 4 and Figure 5). The maxillary bone had less stress in Group 2 models than in Group 1 models (Table 5). The most stress occurred in Model 1A at 1142.20 MPa (in which a tooth-assisted appliance was used and pterygoid plates were not separated), while the least stress occurred in Model 2C at 368.34 MPa (in which a hybrid-assisted appliance was used and pterygoid plates were separated). In both group models, the highest stress occurred in the models using tooth-supported appliances, bone-supported appliances, and hybrid-supported appliances, respectively (Table 5).

Similar to the stresses observed in the bone tissues, less stress was observed on the teeth in Group 2 models compared to those in Group 1 (Figure 5, Table 6). While the stresses on the teeth were primarily associated with tooth-supported appliances, they were generally higher in hybrid-appliance models, though close to those in models using bone-supported appliances. Higher stress was calculated in the models using hybrid-supported appliances, especially in the central and molar teeth regions, compared to models using bone-supported appliances (Figure 5, Table 6). The strain-deformation values in the teeth, presented in Table 7, were also consistent with these results.

## 4. Discussion

We aimed to evaluate two different incisions (the pterygomaxillary junction is separated and not separated) and three different orthodontic appliances (tooth, bone, and hybrid assisted) for treating transverse maxillary deficiency using the FEM to identify the treatment protocol that minimizes the stress on the maxillary bone and teeth. We found that when the pterygomaxillary plates were separated, fewer stresses were observed on the bone and teeth. Although hybrid-supported appliances created less stress on the teeth than tooth-supported appliances and no difference was found between bone-supported appliances, it was found that hybrid-supported appliances created less stress on the bone than the other appliances.

The findings from the FEM offer valuable insights into how different surgical approaches and appliance types might perform in clinical settings. For instance, the stress distributions observed in our models suggest that tooth-supported appliances may lead to localized high-stress areas around the teeth, potentially increasing the risk of complications such as tooth movement or root resorption. On the other hand, bone-supported appliances distribute forces more evenly across the maxilla, potentially reducing the risk of dental complications but possibly leading to discomfort or irritation of soft tissues.

Moreover, the negative effects of tooth-supported appliances, such as increased stress on the periodontal ligament and the possibility of tooth mobility, must be weighed against the potential advantages of more predictable force application. In contrast, bone-supported appliances, while potentially minimizing stress on individual teeth, may require precise fitting and adjustment to avoid soft tissue irritation or bone remodeling complications. These considerations are crucial for translating FEM findings into effective clinical practice, where the choice of appliance must be tailored to the individual patient’s anatomy and clinical needs.

Jensen et al. found that the SARME operation and expansion with orthodontic appliances minimized transverse recurrence by providing more stable results in patients who had reached skeletal maturity [19]. Unlike this study, Chamberland and Proffit [20] found that two-thirds of the skeletal expansion obtained after SARME was permanent and there was no difference between SARME and segmental Le Fort operation in terms of postoperative stabilization. The researchers stated that the SARME operation may be preferred when the maxilla needs to be positioned only in the transverse direction, while segmental Le Fort should be applied when it is necessary to reposition both transversely and antero-posteriorly or vertically [20]. Betts [3] stated that the pterygoid plates should be released to apply the ideal expansion treatment. Zemann et al. [14] performed midpalatal osteotomy in the SARME operation and it was reported that although this provides a more appropriate expansion for the maxilla, less tipping occurs compared to the literature. In our study, while midpalatal osteotomy and lateral corticotomy in zygomatic buttresses were applied to both group models in which SARME operation incisions were created, the separation of pterygoid processes was applied only to Group 2 models and compared with Group 1 models. In the models in which the pterygoid processes were separated, the stresses over the maxilla and teeth were found to be less.

In some studies, it was found that whether the pterygoid plates were separated or not in SARME surgery did not make much difference in terms of the stability of the results [8,21,22,23]. However, separation of the pterygoid plates brings major complications such as skull base fracture, hemorrhage in the internal maxillary artery, or venous plexus [6,11,23,24]. For this reason, while some others suggest the separation of the pterygoid plates, some researchers do not recommend it to avoid fractures in the pterygomaxillary complex [10,22,25,26].

The oral cavity’s complex biomechanical structure makes it nearly impossible to measure forces and stresses on living tissues directly in dentistry, necessitating the use of computational methods like finite element analysis (FEM) to model these interactions. Tehranchi et al. [27] compared the dental and skeletal changes that would occur after the SARME operation in which the tooth-supported and bone-supported orthodontic appliances were used using FEM. In the model obtained from computed tomography, it was found that bone-supported appliances showed a greater skeletal effect compared to tooth-supported appliances and von Mises stresses in the periodontal ligament were reduced by 95% [27]. Similarly, we observed that the most dental displacements, namely deformations, were observed over the molar teeth and the first premolar teeth in models with tooth-supported appliances, and the least in the central teeth. De Assis et al. [13] used FEM to measure the stresses in virtual models. They performed SARME with different surgical osteotomy techniques on the models. Similar to our study, they found that the stresses were less in the models in which the pterygoid plates were separated [13].

The tooth-supported and bone-supported appliances used for maxillary expansion with the RME method were compared using FEM and it was found that bone-supported appliances created more stress than tooth-supported appliances, but were more effective [28]. Although we found that bone-supported appliances caused less stress than tooth-supported appliances, this difference may be due to the support of maxillary expansion with the SARME operation, since the patient in our study was a young adult and would not respond to RME.

Möhlhenrich et al. [29] combined three different surgical techniques with two different bone-supported appliances and performed stress analysis by FEM. In the models, only midpalatal osteotomy, midpalatal osteotomy and lateral corticotomy or midpalatal osteotomy, lateral corticotomy and separation of pterygoid plates were performed and six models were created using two different bone-supported appliances. As a result of the study, less stress and more parallel expansion were observed in the group in which midpalatal osteotomy, lateral corticotomy and pterygomaxillary processes were separated, as observed in our study [29]. Hoxha et al. [30] applied RME and performed finite element analysis using two appliances, bone-supported and hybrid-supported, in the three-dimensional model obtained from the tomography images of a 12-year-old patient. They found that the hybrid-supported appliance applied more force to the teeth and caused more dental displacement [30]. In our study, we found that although the hybrid appliance was similar to the bone-supported appliance in general and caused a little more stress from time to time, it created more stress over the molars and caused more dental movement.

There are some limitations in our study. First, we assumed the bone to be completely homogeneous and the cortical bone to have equal thickness in every region of the maxilla. This simplification was necessary for the FEM model but may not reflect the true variations in bone density and thickness, which could affect stress distribution. Second, we only applied vertical forces and cannot consider the variability in the direction of force. This limitation was due to the complexity of modeling multi-directional forces accurately. However, our study focused on comparing different treatment protocols, and these limitations are expected not to considerably affect our comparative findings. The primary objective was to evaluate the relative performance of each protocol under consistent conditions, which remains valid even with this simplification.

In this study, we focused on a specific patient demographic under controlled conditions. However, it is important to consider how these results might differ across various patient groups [31,32,33]. For example, younger patients with more pliable bone structures might exhibit different stress distributions compared to older patients with less bone plasticity. Additionally, patients with lower bone density may respond differently to the applied forces, particularly when using bone-supported appliances. Furthermore, the severity of the maxillary deficiency could significantly influence the outcomes, potentially requiring different surgical approaches or appliance modifications. These variations underscore the importance of individualized treatment planning and suggest that further studies are needed to explore these factors across diverse patient populations.

## 5. Conclusions

We concluded that in cases where the SARME operation is performed, separating the pterygoid plates in accordance with the anatomy and using a bone-supported or hybrid-supported appliance will cause much less stress on the maxillary bone and teeth. However, in the presence of periodontal problems in posterior teeth, bone-supported appliances may be advantageous over hybrid appliances

## Figures and Tables

**Table 1:** Study groups.

Group 1: Midpalatal suture separation and Le Fort I corticotomy
Model 1A: tooth-supported appliance
Model 1B: bone-supported appliance
Model 1C: hybrid-supported appliance
Group 2: Midpalatal suture separation, Le Fort I corticotomy, and pterygomaxillary junction separation
Model 2A: tooth-supported appliance
Model 2B: bone-supported appliance
Model 2C: hybrid-supported appliance

**Table 2:** Force magnitudes applied for two different incisions according to the types of appliances used.

Type of Appliances	Group 1	Group 2
Tooth-supported sppliance	350 N	325 N
Bone-supported appliance	480 N	370 N
Hybrid-supported appliance	415 N	400 N

**Table 3:** Poisson’s ratio and Young’s modulus determining the biomechanical properties of the materials in the study [13,17,18].

Material	Poisson’s Ratio	Young’s Modulus (MPa)
Tooth	0.3	20.000
Cortical bone	0.3	17.500

**Table 4:** The number of elements and nodes of the models.

Models	Number of Elements	Number of Nodes
1A	1140730	1659779
1B	1140730	1659779
1C	1140730	1659779
2A	1025386	1341658
2B	1025386	1341658
2C	1025386	1341658

**Table 5:** Maximum von Mises stress values occurring over bone in Group 1 and Group 2 models (MPa).

	Group 1			Group 2		
	Tooth-Supported Appliance (Model 1A)	Bone-Supported Appliance (Model 1B)	Hybrid-Supported Appliance (Model 1C)	Tooth-Supported Appliance (Model 2A)	Bone-Supported Appliance (Model 2B)	Hybrid-Supported Appliance (Model 2C)
Maximum stress	1142.20	827.05	817.31	654.46	406.26	368.34

**Table 6:** Maximum von Mises stress values occurring over teeth in Group 1 and Group 2 models (MPa).

	Group 1			Group 2		
Tooth Number	Tooth-Supported Appliance (Model 1A)	Bone-Supported Appliance (Model 1B)	Hybrid-Supported Appliance (Model 1C)	Tooth-Supported Appliance (Model 2A)	Bone-Supported Appliance (Model 2B)	Hybrid-Supported Appliance (Model 2C)
11	88.19	62.10	62.15	52.31	44.01	49.09
12	73.01	32.22	23.42	19.81	31.69	17.85
13	73.46	58.42	54.87	52.04	55.86	48.70
14	544.28	50.04	39.42	280.54	76.69	45.03
15	90.95	56.17	56.86	51.27	42.76	36.82
16	398.71	73.44	331.31	145.43	58.24	124.17
17	416.84	253.30	322.63	196.33	154.03	201.46
21	74.20	24.64	30.92	41.52	14.79	17.92
22	96.39	39.54	42.74	36.39	16.51	21.03
23	108.40	39.60	43.43	83.43	30.78	42.60
24	553.20	33.22	37.64	178.39	23.71	26.03
25	91.04	47.62	38.20	58.01	33.26	34.78
26	401.93	87.91	362.73	165.78	39.49	208.22
27	382.54	162.17	248.03	61.91	57.06	65.16

**Table 7:** Strain-deformation values occurring over teeth in Group 1 and Group 2 models (%).

	Group 1			Group 2		
Tooth Number	Tooth-Supported Appliance (Model 1A)	Bone-Supported Appliance (Model 1B)	Hybrid-Supported Appliance (Model 1C)	Tooth-Supported Appliance (Model 2A)	Bone-Supported Appliance (Model 2B)	Hybrid-Supported Appliance (Model 2C)
11	0.0041	0.0033	0.0033	0.0032	0.0028	0.0029
12	0.0039	0.0019	0.0016	0.0013	0.0017	0.0011
13	0.0040	0.0047	0.0043	0.0036	0.0041	0.0034
14	0.0302	0.0038	0.0032	0.0152	0.0053	0.0031
15	0.0049	0.0036	0.0033	0.0032	0.0026	0.0025
16	0.0367	0.0052	0.0319	0.0093	0.0041	0.0079
17	0.0241	0.0172	0.0213	0.0149	0.0119	0.0154
21	0.0039	0.0017	0.0021	0.0024	0.0009	0.0013
22	0.0051	0.0026	0.0028	0.0025	0.0013	0.0016
23	0.0056	0.0024	0.0026	0.0052	0.0016	0.0022
24	0.0284	0.0020	0.0024	0.0123	0.0014	0.0019
25	0.0049	0.0031	0.0026	0.0031	0.0018	0.0019
26	0.0217	0.0056	0.0221	0.0085	0.0026	0.0107
27	0.0247	0.0119	0.0179	0.0038	0.0035	0.0039

## Data Availability

The original contributions presented in this study are included in the article; further inquiries can be directed to the corresponding author.
